# Protocol registration or development may benefit the design, conduct and reporting of dose-response meta-analysis: empirical evidence from a literature survey

**DOI:** 10.1186/s12874-019-0715-y

**Published:** 2019-04-11

**Authors:** Chang Xu, Liang-Liang Cheng, Yu Liu, Peng-Li Jia, Ming-Yue Gao, Chao Zhang

**Affiliations:** 1 Center for Evidence-Based Medicine and Clinical Research, Taihe Hospital, Hubei University of Medicine, Shiyan, 442000 China; 2 Chinese evidence based medicine Center, West China Hospital, Sichuan University, Chengdu, China; 3 School of Public Health, Sun Yat-sen University, Guangzhou, China; 4 Gansu Provincial Maternity and Child-care Hospital, Gansu, China; 5 School of management, Shanxi Medical University, Taiyuan, China; 6 Institute of Child Health, University College London, London, UK

**Keywords:** Protocol registration, Protocol development, Dose-response meta-analysis, Methodological quality, Reporting quality

## Abstract

**Background:**

To investigate the prevalence of protocol registration (or development) among published dose-response meta-analyses (DRMAs), and whether DRMAs with a protocol are better than those not.

**Methods:**

Three databases were searched for eligible DRMAs. The modified AMSTAR (14 items) and PRISMA checklists (26 items) were used to assess the methodological and reporting quality, with each item assigned 1 point if it met the requirement or 0 if not. We matched (1,2) DRMAs with registered or published protocol to those not, by region and publication years. The summarized quality score and compliance rate of each item were compared between the two groups. Multivariable regression was employed to see if protocol registration or development was associated with total quality score.

**Results:**

We included 529 DRMAs, with 45 (8.51%) completed protocol registration or development. We observed a higher methodological score for DRMAs with protocol than the matched controls (9.47 versus 8.58, *P* <  0.01); this embodied in 4 out of 14 items of AMSTAR [e.g., Duplicate data extraction (rate difference, RD = 0.17, 95% CI: 0.04, 0.30; *P* = 0.01). A higher reporting score (cubic transformed) for DRMAs with protocol than the matched controls was also observed (11,875.00 versus 10,229.53, *P* <  0.01); which embodied in 6 out of 26 items of PRISMA [e.g. Describe methods for publication bias (RD = 0.08, 95% CI: 0.01, 0.14; *P* = 0.02)]. Regression analysis suggested positive association between protocol registration or development and total reporting score (*P* = 0.012) while not for methodological score (*P* = 0.87).

**Conclusions:**

Only a small proportion of DRMAs completed protocol registration or development, and those with protocol were better reported than those not. Protocol registration or development is highly desirable.

**Electronic supplementary material:**

The online version of this article (10.1186/s12874-019-0715-y) contains supplementary material, which is available to authorized users.

## Background

Systematic reviews and meta-analyses represent the highest level of evidence in the hierarchy of clinical evidence of medicine, and becoming increasingly popular in multiple domains [1–3]. Since 1986, over 30,000 systematic reviews and meta-analyses reports have been published, and the number continued to increase rapidly [4]. The extensive growth however partly contributes to overlapping production of systematic reviews and unnecessary duplications [5–7]. The quality and value of such works has been questioned by the scientific community for causing confusions and research waste [4, 8].

Great efforts have been expended to solve the problem [9, 10]. One important work is the launch of the international prospective registry platform (PROSPERO, https://www.crd.york.ac.uk/prospero/) of systematic reviews. Since then, a large number (more than 20,000 by 2016) of registrations were recorded and opened to the public [11]. Another important effort was the development of guidelines for systematic reviews, such as the PRISMA and PRISMA-P statements, which provided guidance for the conducting and reporting of systematic reviews and their protocols [10, 12, 13]. The prospective registration or publication of protocol of systematic reviews a priori is expected to be a valid way to reduce aforementioned research waste [14, 15]. This approach ensures a carefully documented plan before the systematic review starts [12, 13] and allows researchers to see which questions were already registered. It may be beneficial to the quality for the further implementation and reporting.

Dose-response meta-analysis (DRMA) is a type of meta-analysis investigating the dose-response relationship between an exposure and outcome of interest [16, 17]. It becomes popular since Greenland and Orsini published the classical generalized least squares for trend method [16, 17]. Given the aforementioned advantages, adherence to the established checklists for registration or development of protocol may play an important role for the quality of DRMA. This is essential that high quality and informative evidence is the foundation for healthcare decision. Nevertheless, no study had ever investigated the influence of this approach on DRMAs.

We conducted a cross-sectional study by collecting published DRMAs to investigate the prevalence of registration or development of protocol, and whether DRMAs with protocol registration or development were better than those not. The significances of current research are: 1) to verify the importance and realistic value of registration of systematic reviews and meta-analyses; 2) to strengthen the awareness of evidence quality for decision makers and guideline developers; 3) to enhance attention of registration for systematic review and meta-analysis authors.

## Methods

We constructed our research as the following sections: we first searched the main literature databases for eligible DRMAs; the registration (or protocol) information was extracted and the eligible DRMAs were categorized into those registered (or with a protocol) and those not. A matching procedure was applied for DRMAs with protocol registration or development and those not to balance the baseline characteristics. The methodological and reporting quality were then assessed and compared between the registered (or with a protocol) and the matched DRMAs.

### Eligibility criteria

We included dose-response meta-analysis (DRMA) of binary outcomes, without limitation on the design of original studies that included in a DRMA. A dose-response meta-analysis was defined as a form of meta-analysis that combines dose-response relationship between an independent variable (e.g. sleep duration) and an outcome variable (e.g. all-cause mortality) from similar studies [16–20]. This requires a within study dose-response relationship so that the traditional meta-regression were not covered [21]. To distinguish meta-analysis and pooled analysis, the term “meta-analysis” should contains thorough systematic review components, which has been defined by the Cochrane handbook [1].

We did not include DRMA of continuous outcomes as very few such studies were available. We also excluded the following reports: studies specifically investigating methodology of such analyses; analyses based on individual patient data; and unpublished reports, conference abstracts and other forms of short reports (e.g. brief reports, letters).

### Literature search

Medline (Ovid), Embase (Ovid), and Wiley online Library were searched from 1st-Jan-2011 to 31st-Dec-2015 without any language limitation (conducted in 31st-Dec-2015). An updated search was conducted in 1st-Aug-2017. We restricted such time period because very few DRMAs published before 2011. Studies were not included if it was published ahead of print. The search strategy was presented in appendix file (Additional file 1: Appendix 1). The process of literature search was conducted by one experienced author (X.C).

### Study selection process

The study selection was conducted by two experienced, methods-trained authors (X.C and L.Y). One author (X.C) is the primary co-developer of the robust error meta-regression (REMR) model for dose-response meta-analysis [18]. The two authors initially screened titles and abstracts to exclude reports that explicitly failed to meet eligibility criteria. Then, they read the full texts to check against the eligibility criteria independently. Afterwards, they independently collected information from each eligible study, and assessed methodological and reporting quality of included DRMAs. For each of the step, the authors carefully cross-checked the collected data. Any disagreements were resolved through discussion.

### Definition of scientific quality

The scientific quality of a systematic review or meta-analysis generally contains two components, which were the methodological quality and the reporting quality [10, 22]. The former one refers to the internal validity that reflects the design and conduct of a systematic review or meta-analysis, while the later reflects the reporting.

### Data collection and quality assessment

We collected the following background information from each eligible DRMA: name of first author, departments of authors, region of first author, author numbers, year of publication, research topics, journal name, and funding information. We determined that a DRMA registered or with a protocol, if they provided a registration ID, an attachment protocol, or a web linkage of the protocol, which has been described in PRISMA checklist [10]. If a DRMA was registered at PROSPOERO and clinicatrials.gov, we treated it as PROSPERO which is specific for registration of systematic reviews. Those DRMAs claiming registered or developed a protocol, while failed to provide any details and cannot be obtained by our further attempt (searching the supplementary file and the websites of the institution of the first and corresponding author), were not treated as such.

Following a common approach [22–24], we used the AMSTAR 1.0 checklist (Assess Methodological Quality of Systematic Reviews, AMSTAR) to assess the methodological quality and the PRISMA checklist (Preferred Reporting Items for Systematic Reviews and Meta-Analyses, PRISMA) to assess the reporting quality of included DRMAs [10, 25, 26]. We used AMSTAR 1.0 because version 2.0 was yet to be released in that period. For AMSTAR, we made slight modifications by disaggregating four items. For example, we changed the item “*was there duplicate study selection and data extraction?*” into two questions: *was there duplicate study selection?* and *was there duplicate data extraction?*. We removed the item “*was an a priori design provided?*”, because this item specifically addresses the issue about our exposure of interest. These modifications resulted in 14 items (Additional file 1: Appendix 2).

For PRISMA, we also made slight modifications by removing the item “*protocol and registration*” as this item is our exposure of interest. As a result, the modified PRISMA checklist contained 26 items (Additional file 1: Appendix 2).

### Matching between registered and control groups

Given the significantly imbalanced distribution in the geographic region and year of publication between DRMAs with protocol registration or development versus those not, we used propensity score matching method, with nearest neighbor approach, in a 1:2 ratio of the two types of DRMAs [27]. We divided geographic region as Asian and Non-Asian (European, America, and Australia) for the matching since previous literatures demonstrated that meta-analyses and randomized controlled trials by author from Asian tends to poorly conducted [4, 28]. We used 1:2 ratio due to the consideration of both the available sample size and the optimal matching (i.e. a 1:1 ratio may result in low power and a 1:3 ratio or more may lead to over-matching). The details of the matching process can be found in Additional file 1: Appendix 3.

### Statistical analysis

We assigned one point to each of the items if the DRMA met the requirement or zero if not (we treated “unclear” as not), both for the AMSTAR and PRISMA checklists [21]. Thus the total scores were 14 and 26 points, respectively. The compliance rate of each of the item was used to show the extent of those DRMAs met methodological and reporting checklists.

In order to examine if the DRMAs with protocol registration or development would perform better on methodological and reporting quality than those not, we first compared the mean quality scores for AMSTAR and PRISMA between the registered and control group. We used *t* test for the comparison if the scores were normally distributed. Otherwise, we transformed the scores by the method of ladder of powers [29], which would suggest a best approach to data transformation. The test for normality (Skewness-Kurtosis test) suggested that the AMSTAR total score was normally distributed (*P* = 0.37), but not the total score for PRISMA (*P* <  0.01). We subsequently used the cubic transformation for PRISMA total (normality test *P* = 0.32). The compliance rate of each item was compared by *t* test and measured with rate difference (RD).

Multivariable regression analysis was used to see if protocol registration or development was associated with better methodological and reporting quality after adjusting any imbalanced basic characteristics. Considering the potential influence of journals, a robust variance by treating each journal as a cluster, was added to address clustering (on the quality) of papers published in the same journal. All the analyses were conducted using R 3.4.2 and Stata 14.0 software with *p <* 0.05 as statistical significant.

## Results

**Fig. 1:**
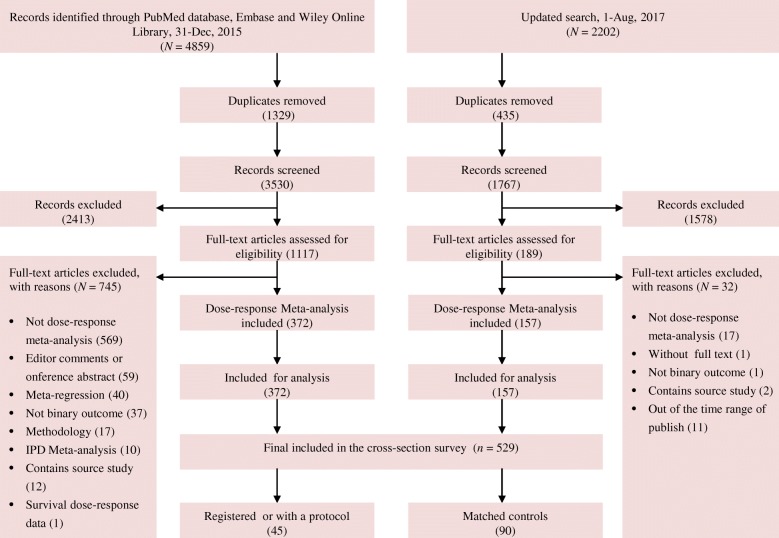
The flow chart of literature screen

The search initially identified 7061 records. After exclusion of duplicate reports and screening of abstracts, 1306 were potentially eligible. Reading full texts against the eligibility criteria finally identified 529 eligible DRMAs (Fig. 1). Of these 529 DRMAs, 45 (8.51%) registered or developed a protocol. The proportion of protocol registration or development were 17.14, 18.18, 7.14, 5.13, 3.33, 5.88, and 16.67% from 2011 to 2017.

Among the 45 studies, 21 were funded by the World Cancer Research Fund UK with study protocols reported at the official website (https://www.wcrf-uk.org/), 19 were registered at the PROSPERO database (https://www.crd.york.ac.uk/prospero/), 3 provided a supplemented protocol, one on website (https://www.gov.uk/government/groups/scientific-advisory-committee-on-nutrition), and one was registered at clinicaltrials.gov (https://clinicaltrials.gov/). When categorized by country, 26 were from UK, 7 from China, 3 from South Korea, 2 from Canada, 2 from Finland, 2 from Germany, 2 from Sweden, and 1 from the US. By propensity score matching, we included 90 DRMAs for comparison (Additional file 1: Appendix 4).

Table 1 presented the basic characteristics of the two groups. The number of authors and proportion of using reporting checklists in the group of DRMAs with protocol registration and development were significant higher than the matched control group. Other basic variables were balanced for the two groups.

**Table 1:** Characteristics of DRMAs with protocol registration or development and matched controls

Basic information	Total sample (*N* = 135)		*P* value
	Registered (*n* = 45)	Matched (*n* = 90)	
Author number [median (IQR)]	8 (7 to 9)	5 (3 to 8)	< 0.01
> = 6	38 (84.44%)	43 (48.78%)	
< 6	7 (15.56%)	47 (52.22%)	
Regions*			
Asian	10 (22.22%)	20 (22.22%)	Reference
European	32 (71.11%)	44 (48.89%)	≈1
America	3 (6.67%)	25 (27.78%)	
Australia	0 (0.00%)	1 (2.22%)	
Database searched [median (IQR)]	3 (2 to 7)	2 (2 to 3)	0.52
> = 2	36 (80.00%)	76 (84.44%)	
< 2	9 (20.00%)	14 (15.56%)	
Funding			0.88
Yes	38 (84.44%)	63 (70.00%)	
No	4 (8.89%)	6 (6.67%)	
Not reported	3 (6.67%)	21 (23.33%)	─
Journal type			0.60
General journal	7 (15.56%)	11 (12.22%)	
Specialist journal	38 (84.44%)	79 (87.78%)	
Publish year*			0.6
2011–2013	18 (40.00%)	32 (35.56%)	
2014–2016	15 (33.33%)	38 (42.22%)	
2017~	12 (26.67%)	20 (22.22%)	
Use of reporting checklist			
Yes	36	56	0.02
No	9	34	
The score of AMSTAR [median (IQR)]	9 (8 to 11)	9 (7 to 10)	─
The score of PRISMA [median (IQR)]	22 (22 to 23)	22 (21 to 24)	─

A general journal means it published articles on all areas (e.g. plos one) or focus on whole medicine area (e.g. BMJ open). For specialist journal, we treat it as those publish articles only on a certain type of disease (e.g. cancer) or a certain body system (e.g. urology)

*IQR* Interquartile range

*Regions (Asian versus Non-Asian) and publish year were matched variables by propensity score

### Methodological quality between registered and matched group

**Fig. 2:**
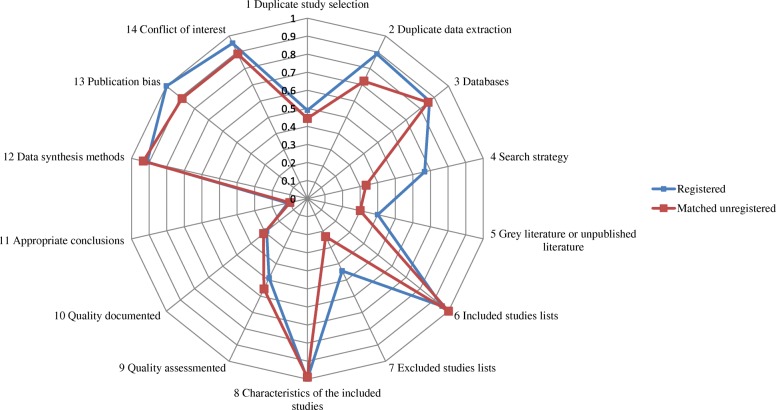
The radar chart of item-based compliance rate for methodological quality

Figure 2 outlined the methodological status of the two groups. In the group of DRMAs with protocol registration or development, the median score of AMSTAR was 9 (first to third quartile: 8, 11), and in the group of matched controls the median score was 9 (first to third quartile: 7, 10). The mean score of DRMAs with protocol registration or development [9.47; Standard deviation (SD): 0.29)] was higher (*P* <  0.01) than the matched group (8.58, SD, 0.20).

For each methodological item, a higher rate of DRMAs with protocol registration or development used duplicate data extraction (RD = 0.17, 95%CI: 0.04, 0.30; *P* = 0.01), documented the search strategy (RD = 0.33, 95% CI: 0.17, 0.50; *P* <  0.01), listed the studies of excluded (RD = 0.21, 95% CI: 0.04, 0.38; *P* = 0.02), and assessed the publication bias (RD = 0.11, 95% CI: 0.04, 0.18; *P* <  0.01) than the matched control group (Table 2).

**Table 2:** The item-based comparison on the quality of registered (or with a prior protocol) DRMAs and matched controls

Quality Checklist	Registered	Matched control	Rate difference	*P* value
Modified AMSTAR				
Item 1: Was there duplicate study selection?	22/45	40/90	0.04 (− 0.13, 0.22)	0.63
Item 2: Was there duplicate data extraction?	40/45	65/90	**0.17 (0.04, 0.30)**	0.01
Item 3: Was there at least two database searched?	39/45	77/90	0.01 (− 0.11, 0.13)	0.86
Item 4: Was there any search strategy documented?	30/45	30/90	**0.33 (0.17, 0.50)**	< 0.01
Item 5: Was the status of publication used as an inclusion criterion?	18/45	27/90	0.10 (−0.07, 0.27)	0.25
Item 6: Was a list of studies of included provided?	43/45	90/90	−0.04 (− 0.11, 0.02)	0.20
Item 7: Was a list of studies of excluded provided?	20/45	21/90	**0.21 (0.04, 0.38)**	0.02
Item 8: Were the characteristics of the included studies provided?	45/45	89/90	0.01 (−0.03, 0.05)	0.59
Item 9: Was the scientific quality of the included studies assessed?	22/45	50/90	−0.07 (− 0.25, 0.11)	0.46
Item 10: Was the scientific quality of the included studies documented (only provide total score should be avoided)?	13/45	28/90	−0.02 (− 0.19. 0.14)	0.79
Item 11: Was the scientific quality of the included studies used appropriately in formulating conclusions?	5/45	9/90	0.01 (−0.10, 0.12)	0.84
Item 12: Were the methods used to combine the findings (dose-response) of studies appropriate?	41/45	84/90	−0.02 (− 0.12, 0.08)	0.66
Item 13: Was the likelihood of publication bias assessed?	45/45	80/90	**0.11 (0.04, 0.18)**	< 0.01
Item 14: Was the conflict of interest stated?	43/45	80/90	0.07 (−0.02. 0.16)	0.14
Modified PRISMA				
Item 1 (Title): Identify the report as a systematic review, meta-analysis, or both.	45/45	90/90	0.00 (−0.03, 0.03)	≈ 1
Item 2 (Introduction): Describe the rationale for the review in the context of what is already known.	45/45	90/90	0.00 (−0.03, 0.03)	≈ 1
Item 3 (Introduction): Provide an explicit objective(s) with reference to PICOS principle.	45/45	90/90	0.00 (−0.03, 0.03)	≈ 1
Item 4 (Methods): Specify criteria for eligibility, giving rationale.	43/45	87/90	−0.01 (− 0.08, 0.06)	0.76
Item 5 (Methods): Describe all information sources (e.g. databases) in the search and date last searched.	45/45	90/90	0.00 (−0.03, 0.03)	≈ 1
Item 6 (Methods): Present full electronic search strategy for at least one database.	36/45	32/90	**0.44 (0.29, 0.60)**	< 0.01
Item 7 (Methods): State the process for selecting studies (two stage: title and abstract screen, then the full text).	15/45	21/90	0.10 (−0.06, 0.26)	0.23
Item 8 (Methods): Describe method of data extraction and any processes for obtaining and confirming data.	40/45	71/90	0.10 (−0.03, 0.23)	0.12
Item 9 (Methods): List and define all variables for which data were sought and any assumptions made.	41/45	82/90	0.00 (−0.10, 0.10)	≈ 1
Item 10 (Methods): Describe methods used for assessing risk of bias of individual studies.	26/45	56/90	−0.04 (− 0.22, 0.13)	0.62
Item 11 (Methods): State the principal summary measures (e.g., risk ratio, difference in means).	43/45	80/90	0.07 (−0.02, 0.16)	0.14
Item 12 (Methods): Describe the methods of handling data and combining results of studies.	45/45	90/90	0.00 (−0.03, 0.03)	≈ 1
Item 13 (Methods): Specify any assessment of risk of bias for the pooled evidence (e.g. publication bias).	45/45	83/90	**0.08 (0.01, 0.14)**	0.02
Item 14 (Methods): Describe methods of additional analyses (e.g. sensitivity analysis, meta-regression).	45/45	86/90	0.04 (−0.01, 0.10)	0.11
Item 15 (Results): Give numbers of studies screened, assessed for eligibility, and included in the review, with reasons for exclusions at each stage, ideally with a flow diagram.	41/45	83/90	−0.01 (− 0.11, 0.09)	0.83
Item 16 (Results): For each study, present characteristics for which data were extracted.	44/45	90/90	−0.02 (− 0.08, 0.03)	0.42
Item 17 (Results): Present data on risk of bias of within each study (study level).	22/45	45/90	−0.01 (− 0.19, 0.17)	0.90
Item 18 (Results): Present summery data, effect estimates and confidence intervals for each study.	45/45	88/90	0.02 (−0.02, 0.07)	0.34
Item 19 (Results): Present results of each meta-analysis, with confidence intervals and measures of consistency.	45/45	90/90	0.00 (−0.03, 0.03)	≈ 1
Item 20 (Results): Present results of risk of bias across studies (publication bias, outcome level).	45/45	80/90	**0.11 (0.04, 0.18)**	< 0.01
Item 21 (Results): Give results of additional analyses, if done.	45/45	87/90	0.03 (−0.02, 0.08)	0.20
Item 22 (Discussion): Summarize the main findings including the strength of evidence for each main outcome.	45/45	88/90	0.02 (−0.02, 0.07)	0.34
Item 23 (Discussion): Discuss limitations at study and outcome level	44/45	80/90	**0.09 (0.01, 0.17)**	0.03
Item 24 (Discussion): Provide a general interpretation of the results and implications for future research.	45/45	90/90	0.00 (−0.03, 0.03)	≈ 1
Item 25 (Funding): Describe sources of funding and other support for the systematic review.	43/45	70/90	**0.18 (0.07, 0.28)**	< 0.01

Rate difference was the absolute difference of the adherence rate of the two groups, and the statistical inference was conducted by *t* test. Those in bold were statistically significant (*p* < 0.05)

Simultaneously, a higher rate of DRMAs with protocol registration or development used the status of publication as an inclusion criterion (RD = 0.10, 95% CI: -0.07, 0.27; *P* = 0.25) and stated the conflicts of interests (RD = 0.07, 95% CI: -0.02, 0.16; *P* = 0.14) than matched control group, though statistically insignificant. However, for scientific quality assessment (risk of bias), a higher proportion was observed among the DRMAs in control group (RD = − 0.07, 95% CI: -0.25, 0.11; *P* = 0.46). There were no obvious rate differences for other items (Table 2).

### Reporting quality between registered and matched control group

**Fig. 3:**
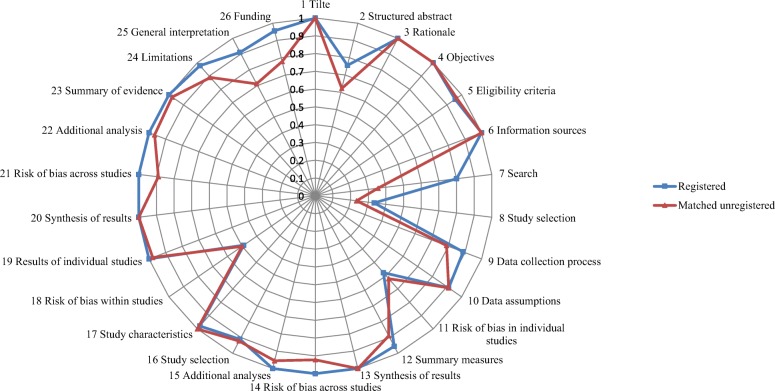
The radar chart of item-based compliance rate for reporting quality

Figure 3 outlined the reporting status of the two groups. In the group of DRMAs with protocol registration or development, the median score of PRISMA was 22 (first to third quartile: 22, 23), and in the group of matched controls the median score was 22 (first to third quartile: 21, 24). The mean cubic transformed score on group of DRMAs with protocol registration or development (12,967.13, SD: 2480.492) was (*P* <  0.01) higher than the matched control group (10,848.88, SD: 3224.55).

For each reporting item, we found a higher rate of DRMAs with protocol registration or development presented full electronic search strategy for at least one database (RD = 0.44, 95% CI: 0.29, 0.60; *P* <  0.01), described methods for publication bias (RD = 0.08, 95% CI: 0.01, 0.14; *P* = 0.02), presented the risk of bias across studies (i.e. publication bias, RD = 0.11, 95% CI: 0.04, 0.18; *P* < 0.01), discussed limitations at study and outcome level (RD = 0.09, 95% CI: 0.01, 0.17; *P* = 0.03), provided a general interpretation of the results (RD = 0.20, 95% CI: 0.08, 0.33; *P* <  0.01), and described sources of funding and other support (RD = 0.18, 95% CI: 0.07, 0.28; *P* <  0.01) than the matched controls (Table 2).

Other items with higher compliance rate among DRMAs with protocol registration or development were: state the process of selecting studies (RD = 0.10, 95% CI: -0.06, 0.26; *P* = 0.23), describe method of data extraction (RD = 0.10, 95% CI: -0.03, 0.23; *P* = 0.12), and state the principal summary measures (RD = 0.07, 95% CI: -0.02, 0.16; *P* = 0.14). No obvious rate difference was observed on other items (Table 2).

### Multivariable regression analysis

After adjusting the unbalanced covariates and clustering on journal in the multivariable regression model, we found that protocol registration or development in priori was significant associated with better reporting quality (β= 1446.66, *P* = 0.012) while not associated with methodological quality (β= 0.06, *P* = 0.88). A post hoc sensitivity analysis was conducted by excluding 3 DRMAs only provided a supplemented protocol (as well as the 6 matched DRMAs), considering that supplemented protocol does not ensure that it was developed a priori. The results did not showed substantial changes [reporting quality (β= 1366.186, *P* = 0.031); methodological quality (β= − 0.03; *P* = 0.93)].

## Discussion

To the best of our knowledge, this is the first research described the proportion of protocol registration (or development) of dose-response meta-analyses (DRMAs) as well as the first research compared the methodological and reporting quality of DRMAs with protocol registration or development to those not. In this study, we found that only 8.51% of the published DRMAs registered or provided a protocol, while these DRMAs have higher compliance rate on some methodological/reporting items and better total reporting quality than those without a protocol.

There were some differences of our results to an earlier research [24]. In the study, the quality of Cochrane systematic reviews (all requires a protocol) and reviews published in paper-based journals were compared [24], and higher methodology rigors (instead of reporting) of Cochrane systematic reviews were observed. A more recent survey suggested that prospective registration may improve the overall methodological quality of systematic reviews of randomized controlled trial [30]. However, in our study, we only observed a significant improvement on total reporting quality while not for methodological quality. This is likely due to the different types of meta-analysis we aimed at.

Based on our results, for DRMAs failed to provide a protocol, the under complied methodological items were: few of them employed duplicate data extraction, documented the search strategy, documented the scientific quality, and listed the studies of excluded. While the under complied reporting items were: few of them presented full electronic search strategy, stated the process of for selecting studies, described methods for assessing risk of bias within each study, and presented the data on risk of bias of within each study. For both groups, the scientific quality of included studies were seldom documented, this subsequently result in the failing of applying the scientific quality to formulate conclusions.

The AMSTAR and PRISMA checklists were widely used to assess methodological and reporting quality of systematic reviews and meta-analyses [30, 31]. The AMSTAR was first released in 2007 that was of two years earlier than the PRISMA checklist (released in 2009) [10, 26]. In our research, the PRISMA checklist was generally better complied than the AMSTAR (See Figs. 2 and 3). This may partly due to the promotion of academic journals, as a large number of journals require the submissions should in accordance with the PRISMA checklist. The phenomenon however may reflect a serious issue — the lack of focus on methodology of systematic reviews and meta-analyses. Our findings suggested that even for DRMAs with a protocol, many of the methodological items were poorly conducted. It is expected more attention should be paid to the methodology of systematic reviews and meta-analyses in the future.

The findings of current research have verified the importance of protocol development or registration of systematic reviews and meta-analyses. Protocol may benefits healthcare decision in two aspects. First, evidence is the key body for evidence based practice, and a prior registration of systematic reviews and meta-analyses benefits the evidence production that further help to better healthcare decision. In addition, a prior registration may make sense to improve the efficiency of decision making given the potential role on reducing overlapped evidence production.

There were some strengthens of current research that help to enhance the credibility of our results. We adopted a comprehensive and up-to-date search, where we almost included all potential published DRMAs during the past 7 years. We employed the 1:2 matching by propensity score method and multiple regression to control the potential confounders on our results. We blinded the process of quality assessment to avoid objective bias. However, several limitations should not be ignored. In this article, we did not take account for the correlation between AMSTAR and PRISMA. Actually, these two checklists are highly correlated since some items of them were overlapped or even the same. We did not mix them together because we believe that comparing methodological quality and reporting quality separately is of more practical significance. In addition, AMSTAR and PRISMA are currently the optimal tools to reflect the quality of systematic reviews, yet it is impossible for them to cover all of the domains. Our results highly rely on the validity and reliability of the two checklists. Third, we failed to distinguish the potential heterogeneity among different registration methods (e.g. PROSPERO, documented protocol) due to the limited numbers of DRMAs with protocol. Fourth, the information of protocol registration or development was collected based on the reporting of these published DRMAs, it is possible that a small amount of DRMAs may publish a protocol in advance but not mentioned in the final context, which would lead to selective bias for our results. Fifth, for DRMAs with protocol registration or development, many of which shared the same first author that may influence the representativeness.

## Conclusions

Based on the current survey, only a small proportion of published DRMAs completed protocol registration or development and the methodological quality of these DRMAs is suboptimal. DRMAs with a protocol had better reporting quality and higher compliance rate of several methodological quality related items than those not. Therefore, protocol registration or development may benefit for evidence based practice that is highly desirable for all DRMAs.

## Additional file

Additional file 1: Search strategy, modified checklist for quality assessment, R code and hist plots of propensity score matching, and list of included DRMAs. (DOCX 122 kb)

## Abbreviations

AMSTAR: Assess Methodological Quality of Systematic Reviews
DRMA: Dose-response meta-analysis
PRISMA: Preferred Reporting Items for Systematic Reviews and Meta-Analyses
PRISMA-P: Preferred reporting items for systematic review and meta-analysis protocols
RD: Rate difference
REMR: Robust error meta-regression
SD: Standard deviation
