# Supplementary material for: Protocol registration or development may benefit the design, conduct and reporting of dose-response meta-analysis: empirical evidence from a literature survey
Source: BMC Med Res Methodol. 2019 Apr 11;19:78. doi: 10.1186/s12874-019-0715-y (PMC6460643; doi:10.1186/s12874-019-0715-y)
Supplement: Supplementary file 1 — Search strategy, modified checklist for quality assessment, R code and hist plots of propensity score matching, and list of included DRMAs. (DOCX 122 kb) [file 12874_2019_715_MOESM1_ESM.docx]

**Appendix 1. Search strategy**

**Ovid MEDLINE(R) In-Process & Other Non-Indexed Citations and Ovid MEDLINE(R) 1946 to Present (2011 to 2015-Dec-31)**

| **Items** | **Searches** | **Results** | **Search Type** |
| --- | --- | --- | --- |
| 1 | Dose Response Relationship*/exp {No Related Terms} | 473 | Basic |
| 2 | Dose-response association*/exp {No Related Terms} | 9478 | Basic |
| 3 | Dose-effect*/exp {No Related Terms} | 6287 | Basic |
| 4 | or/1-3 | 16162 | Advanced |
| 5 | Meta-analysis/exp {No Related Terms} | 19789 | Basic |
| 6 | ''Pool? Analysis'' {No Related Terms} | 8322 | Basic |
| 7 | Systematic review*/exp {No Related Terms} | 15451 | Basic |
| 8 | or/5-7 | 39299 | Advanced |
| 9 | 4 and 8 | 120 | Advanced |
| 10 | (''dose-response meta-analysis'' or ''non-linear meta-regression'' or ''meta-analysis of prospective studies'' or ''meta-analysis of cohort studies'' or ''meta-analysis of observational studies'').tw,ot. | 1613 | Advanced |
| 11 | 9 or 10 | 1730 | Advanced |
| 12 | animals/ not (Humans/ and Animals/) {No Related Terms} | 10028 | Basic |
| 13 | 11 not 12 | 1730 | Advanced |
| **14** | **13 and 2011:2015.(sa_year).** | **1381** | **Advanced** |

**Embase database**

| **No.** | **Query** | **Results** |
| --- | --- | --- |
| #19 | **#18** AND [embase]/lim | **2,474** |
| #18 | **#17** AND (**'evidence based medicine'**/de OR **'human'**/de OR **'meta analysis'**/de OR **'meta analysis (topic)'**/de OR **'systematic review'**/de) AND (**2011**:py OR **2012**:py OR **2013**:py OR **2014**:py OR **2015**:py) | **2,598** |
| #17 | **#10** OR **#16** | **6,450** |
| #16 | **#11** OR **#12** OR **#13** OR **#14** OR **#15** | **1,832** |
| #15 | **'meta-analysis of observational studies'** | **1,008** |
| #14 | **'meta-analysis of cohort studies'** | **290** |
| #13 | **'meta-analysis of prospective studies'** | **375** |
| #12 | **'non-linear meta-regression'** | **1** |
| #11 | **'dose-response meta-analysis'** | **262** |
| #10 | **#5 AND #9** | **4,788** |
| #9 | **#6 OR #7 OR #8** | **164,355** |
| #8 | **'systematic review'/exp** | **101,893** |
| #7 | **'pool? analysis'** | **17** |
| #6 | **'meta analysis'**/exp | **102,438** |
| #5 | **#1** OR **#2** OR **#3** OR **#4** | **372,838** |
| #4 | **'dose-effects'** | **1,534** |
| #3 | **'dose-effect'**/exp | **371,631** |
| #2 | **'dose-response association'** | **485** |
| #1 | **'dose response relationship'**/exp | **371,631** |

**Wiley Online Library (2011~2015)** *1004 results were presented*

(''dose-response meta-analysis'' or ''non-linear meta-regression'' or ''meta-analysis of prospective studies'' or ''meta-analysis of cohort studies'' or ''meta-analysis of observational studies'') in Abstract NOT animal* in All Fields between years 2011 and 2015

**Appendix 2. Items of Modified AMSATR and PRISMA**

| **Modified AMSTAR** |
| --- |
| Item 1: Was there duplicate study selection? |
| Item 2: Was there duplicate data extraction? |
| Item 3: Was there at least two database searched? |
| Item 4: Was there any search strategy documented (only provide total score should be avoid)? |
| Item 5: Was the status of publication used as an inclusion criterion? |
| Item 6: Was a list of studies of included provided? |
| Item 7: Was a list of studies of excluded provided? |
| Item 8: Were the characteristics of the included studies provided? |
| Item 9: Was the scientific quality of the included studies assessed? |
| Item 10: Was the scientific quality of the included studies documented? |
| Item 11: Was the scientific quality of the included studies used appropriately in formulating conclusions? |
| Item 12: Were the methods used to combine the findings (dose-response) of studies appropriate? |
| Item 13: Was the likelihood of publication bias assessed? |
| Item 14: Was the conflict of interest stated? |
| **Modified PRISMA** |
| Item 1 (Title): Identify the report as a systematic review, meta-analysis, or both. |
| Item 2 (Abstract): Structured abstract. |
| Item 3 (Introduction): Describe the rationale for the review in the context of what is already known. |
| Item 4 (Introduction): Provide an explicit objective(s) with reference to PICOS principle. |
| Item 5 (Methods): Specify criteria for eligibility, giving rationale. |
| Item 6 (Methods): Describe all information sources (e.g. databases) in the search and date last searched. |
| Item 7 (Methods): Present full electronic search strategy for at least one database. |
| Item 8 (Methods): State the process for selecting studies (two stage: title and abstract screen, then the full text). |
| Item 9 (Methods): Describe method of data extraction and any processes for obtaining and confirming data. |
| Item 10 (Methods): List and define all variables for which data were sought and any assumptions made. |
| Item 11 (Methods): Describe methods used for assessing risk of bias of individual studies. |
| Item 12 (Methods): State the principal summary measures (e.g., risk ratio, difference in means). |
| Item 13 (Methods): Describe the methods of handling data and combining results of studies. |
| Item 14 (Methods): Specify any assessment of risk of bias for the pooled evidence (e.g. publication bias). |
| Item 15 (Methods): Describe methods of additional analyses (e.g. sensitivity analysis, meta-regression). |
| Item 16 (Results): Give numbers of studies screened, assessed for eligibility, and included in the review, with reasons for exclusions at each stage, ideally with a flow diagram. |
| Item 17 (Results): For each study, present characteristics for which data were extracted. |
| Item 18 (Results): Present data on risk of bias of within each study (study level). |
| Item 19 (Results): Present summery data, effect estimates and confidence intervals for each study. |
| Item 20 (Results): Present results of each meta-analysis, with confidence intervals and measures of consistency. |
| Item 21 (Results): Present results of risk of bias across studies (publication bias, outcome level). |
| Item 22 (Results): Give results of additional analyses, if done. |
| Item 23 (Discussion): Summarize the main findings including the strength of evidence for each main outcome. |
| Item 24 (Discussion): Discuss limitations at study and outcome level |
| Item 25 (Discussion): Provide a general interpretation of the results and implications for future research. |
| Item 26 (Funding): Describe sources of funding and other support for the systematic review. |

**Appendix 3. R code and hist plots of propensity score matching.**

*library(MatchIt)*

*mydata <- read.csv ("C:/ Users /desktop/Quality/data.csv")*

*attach mydata*

*mydata[1:10,]*

*set.seed(1000) #very important to keep data set stable*

*m.out = matchit(Regis ~ year + Area, data = mydata, method = "nearest", ratio = 2, distance = "logit")*

*summary(m.out)*

*plot(m.out, type = "hist")*

**Figure S1.** The hist plot of 1:2 matching (well-matched).


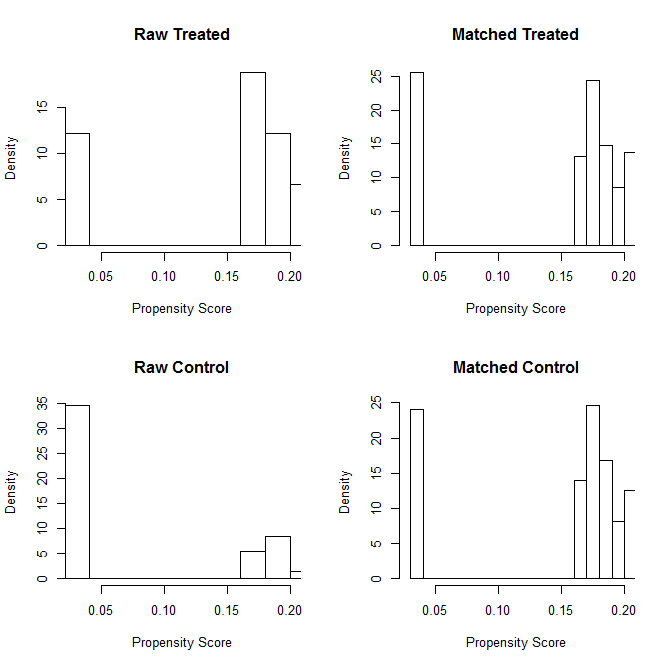


**Figure S2.** The hist plot of 1:3 matching (obviously over-matching).


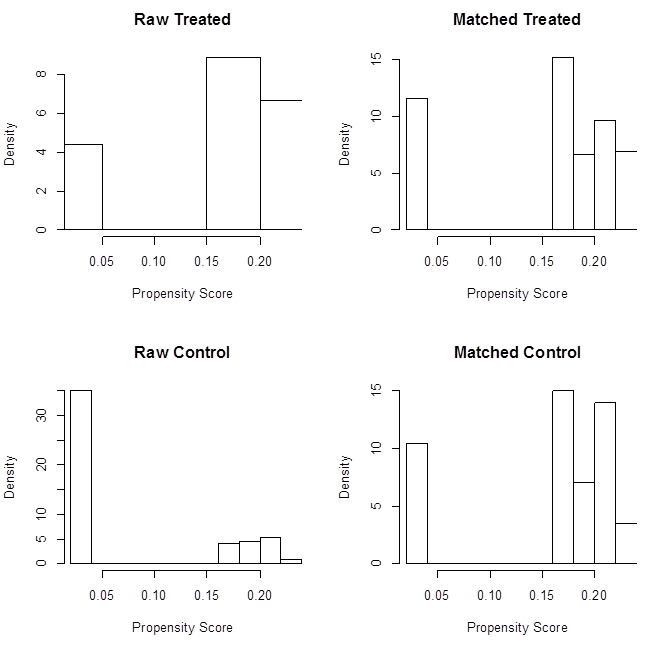


**Appendix 4. Registered and matched dose-response meta-analysis.**

| **First author** | **Country** | **Citations** | **Registration** |
| --- | --- | --- | --- |
| ***Registered or with a protocol (n=45)*** | | | |
| Abar L | UK | Cancer Med. 2016 Aug;5(8):2069-83. | Website (WCRF) |
| Aune | Norway/UK | Am J Clin Nutr. 2015 Jan;101(1):87-117. | Website (WCRF) |
| Aune D | UK | Cancer Causes Control. 2012 Aug;23(8):1213-22. | Website (WCRF) |
| Aune D | UK | Am J Clin Nutr. 2012 Aug;96(2):356-73. | Website (WCRF) |
| Aune D | UK | Ann Oncol. 2012 Jun;23(6):1394-402. | Website (WCRF) |
| Aune D | UK | Ann Oncol. 2012 Apr;23(4):843-52. | Website (WCRF) |
| Aune D | UK | Int J Cancer. 2015 Apr 15;136(8):1888-98. | Website (WCRF) |
| Aune D | UK | Ann Oncol. 2015 Aug;26(8):1635-48. | Website (WCRF) |
| Aune D | UK | Int J Cancer. 2015 Apr 15;136(8):1888-98. | Website (WCRF) |
| Aune D | UK | Ann Oncol. 2012 Oct;23(10):2536-46. | Website (WCRF) |
| Aune D | UK | Breast Cancer Res Treat. 2012 Jul;134(2):479-93. | Website (WCRF) |
| Aune D | UK | Cancer Causes Control. 2012 Apr;23(4):521-35. | Website (WCRF) |
| Aune D | UK | Ann Oncol. 2012 Jan;23(1):37-45. | Website (WCRF) |
| Aune D | UK | BMJ. 2011 Nov 10;343:d6617. | Website (WCRF) |
| Aune D | UK | Gastroenterology. 2011 Jul;141(1):106-18. | Website (WCRF) |
| Chan DS | UK | PLoS One. 2011;6(6):e20456. | Website (WCRF) |
| Fang X | Sweden | Nutrients. 2016 Nov 19;8(11). pii: E739. | PROSPERO |
| Fang X | Sweden | J Trace Elem Med Biol. 2016 Dec;38:64-73. | Website (WCRF) |
| Gilbert R | UK | Cancer Causes Control. 2011 Mar;22(3):319-40. | PROSPERO |
| Gong F | China | Nutrients. 2017 Apr 20;9(4). pii: E402. | PROSPERO |
| Greenwood DC | UK | Diabetes Care. 2013 Dec;36(12):4166-71. | Website |
| Guo P | China | J Diabetes Complications. 2017 Jan;31(1):58-66. | PROSPERO |
| Han H | China | Nutr J. 2017 May 5;16(1):26. | PROSPERO |
| Han H | China | Oncotarget. 2017 Jul 25;8(30):50164-50173. | PROSPERO |
| Heslehurst N | UK | Obes Rev. 2017 Mar;18(3):293-308. | PROSPERO |
| Hurst R | UK | Am J Clin Nutr. 2012 Jul;96(1):111-22. | Website (WCRF) |
| Ijaz S | Finland | Scand J Work Environ Health. 2013 Sep 1;39(5):431-47. | PROSPERO |
| Jayalath VH | Canada | Am J Clin Nutr. 2015 Oct;102(4):914-21. | clinicaltrials.gov |
| Ju SY | South Korea | Nutrients. 2014 Dec 22;6(12):6005-19. | PROSPERO |
| Ju SY | South Korea | J Clin Endocrinol Metab. 2014 Mar;99(3):1053-63. | PROSPERO |
| Kelly P | UK | Int J Behav Nutr Phys Act. 2014 Oct 24;11:132. | PROSPERO |
| Kennedy OJ | UK | BMJ Open. 2017 May 9;7(5):e013739. | Supplemented protocol |
| Kwon Y | South Korea | PLoS One. 2017 Jan 3;12(1):e0168247. | PROSPERO |
| Kyu HH | USA | BMJ. 2016 Aug 9;354:i3857. | Supplemented protocol |
| Liao WC | China | BMJ. 2014 Jan 2;350:g7371. | Supplemented protocol |
| Livesey G | UK | Am J Clin Nutr. 2013 Mar;97(3):584-96. | PROSPERO |
| Neilson HK | Canada | Menopause. 2017 Mar;24(3):322-344. | PROSPERO |
| Schwingshackl L | Germany | Eur J Epidemiol. 2017 May;32(5):363-375. | PROSPERO |
| Schwingshackl L | Germany | Am J Clin Nutr. 2017 Jun;105(6):1462-1473. | PROSPERO |
| Touvier M | UK | Cancer Epidemiol Biomarkers Prev. 2011 May;20(5):1003-16. | Website (WCRF) |
| Verbeek J | Finland | Saf Health Work. 2017 Jun;8(2):130-142. | PROSPERO |
| Vieira AR | UK | Cancer Med. 2015 Jan;4(1):136-46. | Website (WCRF) |
| Vieira AR | UK | Ann Oncol. 2016 Jan;27(1):81-96. | Website (WCRF) |
| Wang B | China | Eur J Endocrinol. 2014 Nov;171(5):R173-82. | PROSPERO |
| Zhou Q | China | Arch Gynecol Obstet. 2017 Feb;295(2):467-479. | PROSPERO |
| ***Matched unregistered (n=90)*** | | | |
| Abete I | UK | Br J Nutr. 2014 Sep 14;112(5):762-75. | ─ |
| Alexander DD | USA | Eur J Cancer Prev. 2011 Jul;20(4):293-307. | ─ |
| Amadou A | France | Obes Rev. 2013 Aug;14(8):665-78. | ─ |
| Aune D | Norway/UK | BMJ. 2016 Jun 14;353:i2716. | ─ |
| Aune D | Norway/UK | BMC Med. 2016 Dec 5;14(1):207. | ─ |
| Aune D | Norway/UK | Colorectal Dis. 2017 Jul;19(7):621-633. | ─ |
| Aune D | Norway/UK | Eur J Epidemiol. 2017 Mar;32(3):181-192. | ─ |
| Aune D | Norway/UK | NUTR METAB CARDIOVAS.2017 June, 27, 504e517 | ─ |
| Aune D | Norway/UK | Eur J Epidemiol. 2013 Nov;28(11):845-58. | ─ |
| Aune D | UK | Am J Clin Nutr. 2013 Oct;98(4):1066-83. | ─ |
| Bagnardi V | Italy | Ann Oncol. 2011 Dec;22(12):2631-9. | ─ |
| Bellocco R | Italy | Ann Oncol. 2012 Sep;23(9):2235-44. | ─ |
| Bischoff-Ferrari HA | Switzerland | J Bone Miner Res. 2011 Apr;26(4):833-9. | ─ |
| Boyd RA | USA | Clin Transl Sci. 2017 Jul;10(4):260-270. | ─ |
| Burgaz A | Sweden | J Hypertens. 2011 Apr;29(4):636-45. | ─ |
| Burgers AM | Netherland | J Clin Endocrinol Metab. 2011 Sep;96(9):2912-20. | ─ |
| Chen C | China | J Diabetes Investig. 2017 Jul;8(4):480-488. | ─ |
| Chung M | USA | Ann Intern Med. 2011 Dec 20;155(12):827-38. | ─ |
| Dahabreh IJ | USA | Lung Cancer. 2012 May;76(2):150-8. | ─ |
| de Goede J | Netherland | J Am Heart Assoc. 2016 May 20;5(5). | ─ |
| Di Giuseppe D | Sweden | Arthritis Res Ther. 2014 Sep 30;16(5):446. | ─ |
| Ding M | USA | Circulation. 2014 Feb 11;129(6):643-59. | ─ |
| Druesne-Pecollo N | France | Breast Cancer Res Treat. 2012 Oct;135(3):647-54. | ─ |
| Fry JS | UK | BMC Cancer. 2012 Oct 27;12:498. | ─ |
| Gijsbers L | Netherland | Am J Clin Nutr. 2016 Apr;103(4):1111-24. | ─ |
| Greenwood DC | UK | Eur J Epidemiol. 2014 Oct;29(10):725-34. | ─ |
| Grosso G | Italy | Mol Nutr Food Res. 2016 Jan;60(1):223-34. | ─ |
| Grosso G | Italy | Am J Epidemiol. 2017 Jun 15;185(12):1304-1316. | ─ |
| Grosso G | Italy | J Affect Disord. 2016 Nov 15;205:269-281. | ─ |
| Grosso G | Italy | Eur J Epidemiol. 2016 Dec;31(12):1191-1205. | ─ |
| Guo J | UK | Eur J Epidemiol. 2017 Apr;32(4):269-287. | ─ |
| Hu EA | USA | BMJ. 2012 Mar 15;344:e1454. | ─ |
| Je Y | USA | Int J Cancer. 2012 Oct 1;131(7):1700-10. | ─ |
| Jiang R | Germany | Br J Nutr. 2016 Dec;116(12):2115-2128. | ─ |
| Jiang WB | China | Int J Clin Exp Med 2017;10(1):88-96 | ─ |
| Kaluza J | Poland | Stroke. 2012 Oct;43(10):2556-60. | ─ |
| Kanhai DA | Netherland | Obes Rev. 2013 Jul;14(7):555-67. | ─ |
| Karahalios A | Australia | Am J Epidemiol. 2015 Jun 1;181(11):832-45. | ─ |
| Kunutsor SK | UK | Int J Clin Pract. 2015 Jan;69(1):136-44. | ─ |
| Kunutsor SK | UK | Int J Epidemiol. 2014 Feb;43(1):187-201. | ─ |
| Kunutsor SK | UK | Int J Cancer. 2015 Mar 1;136(5):1162-70. | ─ |
| Larsson SC | Sweden | Am J Clin Nutr. 2013 May;97(5):951-7. | ─ |
| Larsson SC | Sweden | Eur J Epidemiol. 2012 Dec;27(12):895-901. | ─ |
| Larsson SC | Sweden | Am J Clin Nutr. 2012 Feb;95(2):362-6. | ─ |
| Larsson SC | Sweden | Br J Cancer. 2012 Jan 31;106(3):603-7. | ─ |
| Larsson SC | Sweden | Am J Epidemiol. 2011 Nov 1;174(9):993-1001. | ─ |
| Larsson SC | Sweden | Stroke. 2011 Oct;42(10):2746-50. | ─ |
| Larsson SC | Sweden | Nutrients. 2015 Sep 11;7(9):7749-63. | ─ |
| Li Z | China | Int J Clin Pharmacol Ther. 2017 Mar;55(3):210-219. | ─ |
| Liu TZ | China | Sleep Med Rev. 2017 Apr;32:28-36. | ─ |
| Liu X | China | Acta Diabetol. 2017 Mar;54(3):223-235. | ─ |
| Liu XM | China | Mol Nutr Food Res. 2017 Jun;61(6). | ─ |
| Lu D | China | Cancer Causes Control. 2014 Nov;25(11):1553-63. | ─ |
| Macis D | Italy | Int J Epidemiol. 2014 Aug;43(4):1226-36. | ─ |
| McLeod DS | USA | J Clin Endocrinol Metab. 2012 Aug;97(8):2682-92. | ─ |
| Mocellin S | Italy | J Natl Cancer Inst. 2017 Mar 1;109(3):1-9. | ─ |
| Mostofsky E | USA | Circ Heart Fail. 2012 Jul 1;5(4):401-5. | ─ |
| Ni CX | China | Nutr Cancer. 2017 Feb-Mar;69(2):211-220. | ─ |
| Pan A | USA | Am J Clin Nutr. 2012 Dec;96(6):1262-73. | ─ |
| Pandey A | USA | Circulation. 2015 Nov 10;132(19):1786-94. | ─ |
| Park M | South Korea | Prev Med. 2014 Aug;65:13-22. | ─ |
| Qin W | China | Cardiovasc Ther. 2017 March;35:e12232 | ─ |
| Roerecke M | Canada | Addiction. 2012 Jul;107(7):1246-60. | ─ |
| Qin Z | China | Atherosclerosis. 2017 Jun;261:1-11. | ─ |
| Salehi M | Iran | Nutr Rev. 2013 May;71(5):257-67. | ─ |
| Samokhvalov AV | Canada | EBioMedicine. 2015 Nov 14;2(12):1996-2002. | ─ |
| Schlesinger S | Germany | PLoS One. 2016 Nov 3;11(11):e0165811. | ─ |
| Soedamah-Muthu SS | Netherland | Am J Clin Nutr. 2011 Jan;93(1):158-71. | ─ |
| Aune D | Norway/UK | Circulation. 2016 Feb 16;133(7):639-49. | ─ |
| Song Y | USA | Diabetes Care. 2013 May;36(5):1422-8. | ─ |
| Tamariz L | USA | Congest Heart Fail. 2011 Jan-Feb;17(1):25-30. | ─ |
| Thomopoulos TP | Greece | Cancer Epidemiol. 2015 Dec;39(6):1047-59. | ─ |
| Threapleton DE | UK | BMJ. 2013 Dec 19;347:f6879. | ─ |
| Vinceti M | USA | J Am Heart Assoc. 2016 Oct 6;5(10). | ─ |
| Wallin A | Sweden | Br J Cancer. 2011 Mar 29;104(7):1196-201. | ─ |
| Wang F | China | Int J Cancer. 2014 Oct 1;135(7):1673-86. | ─ |
| Tamez M | USA | Br J Nutr. 2016 Jun;115(12):2212-8. | ─ |
| Wang J | China | PLoS One. 2017 Feb 23;12(2):e0172631. | ─ |
| Wang S | China | Urol Int. 2014;93(2):220-8. | ─ |
| Ward AM | UK | J Hypertens. 2012 Mar;30(3):449-56. | ─ |
| Weng H | China | Front Physiol. 2017 Jan 23;7:693. | ─ |
| Wu L | USA | Hum Reprod. 2015 Sep;30(9):2234-40. | ─ |
| Wu L | USA | Circ Cardiovasc Qual Outcomes. 2012 Nov;5(6):819-29. | ─ |
| Xu X | China | Eur J Cancer Prev. 2013 Nov;22(6):529-39. | ─ |
| Zeng L | China | Nutrients. 2017 May 15;9(5). pii: E500. | ─ |
| Zhang D | USA | Gynecol Oncol. 2016 Aug;142(2):368-77. | ─ |
| Zhang D | USA | J Matern Fetal Neonatal Med. 2017 Mar;30(6):645-657. | ─ |
| Zhang Z | China | Neurology. 2014 Jul 1;83(1):19-25. | ─ |
| Zheng JS | China | Nutr Cancer. 2013;65(1):1-16. | ─ |
| Zhang Z | USA | Am J Clin Nutr. 2011 Jun;93(6):1212-9. |  |
